# Determination of Statistical Properties of Microtubule Populations

**DOI:** 10.4236/am.2016.713125

**Published:** 2016-08-15

**Authors:** Tyson DiLorenzo, Lee Ligon, Donald Drew

**Affiliations:** 1 Department of Mathematics, Rensselaer Polytechnic Institute, Troy, USA; 2 Department of Biological Sciences and Center for Biotechnology and Interdisciplinary Studies, Rensselaer Polytechnic Institute, Troy, USA

**Keywords:** Microtubule, Image Analysis, Curvature

## Abstract

Microtubules are structures within the cell that form a transportation network along which motor proteins tow cargo to destinations. To establish and maintain a structure capable of serving the cell’s tasks, microtubules undergo deconstruction and reconstruction regularly. This change in structure is critical to tasks like wound repair and cell motility. Images of fluorescing microtubule networks are captured in grayscale at different wavelengths, displaying different tagged proteins. The analysis of these polymeric structures involves identifying the presence of the protein and the direction of the structure in which it resides. This study considers the problem of finding statistical properties of sections of microtubules. We consider the research done on directional filters and utilize a basic solution to find the center of a ridge. The method processes the captured image by centering a circle around pre-determined pixel locations so that the highest possible average pixel intensity is found within the circle, thus marking the center of the microtubule. The location of these centers allows us to estimate angular direction and curvature of the microtubules, statistically estimate the direction of microtubules in a region of the cell, and compare properties of different types of microtubule networks in the same region. To verify accuracy, we study the results of the method on a test image.

## 1. Introduction

Microtubules are part of the cellular cytoskeleton and one of their main functions is to serve as a transportation network within the cell. They are made of two subunits: *α-* and *β*-tubulin. A microtubule is typically formed by thirteen protofilament chains made of these *α-β* heterodimers, arranged into a hollow tube approximately 25 μm in diameter [1]. Molecular motor proteins like kinesin can move along the surface of the microtubule, which allows them to tow cargo like organelles and vesicles to other parts of the cell, giving the cell the ability to distribute its contents according to need. Although the microtubule network is distributed throughout the cell, individual microtubules are dynamic and can depolymerize and repolymerize rapidly. This dynamic behavior allows the cell to change the distribution and organization of the entire network quickly when functions such as cell motility and wound repair demand. For example, when the cell is migrating, many microtubules will reorient or repolymerize toward the direction of cell travel, which allows for the delivery of cargos and material to the active leading edge. One of the most dramatic instances of microtubule networks reorganizing occurs during cell division, where the entire microtubule network is restructured to form the machinery of chromosome segregation.

In the cell, it is typically not necessary for individual microtubules to have a precise subcellular localization. Rather, microtubule networks function normally if they are sufficiently dense and enough segments of microtubules have the appropriate orientation. By segments, we mean parts of a mictrotubule along the entire length of the microtubule. These segments could be defined in several ways. In this study, we consider segments of microtubules between where microtubules overlap or cross.

A current problem in microtubule research is determining appropriate statistical measures that quantify the distribution and organization of microtubule networks. Appropriate statistical measures that describe the overall organization of the microtubule network and how the network changes under different conditions can lead to a better understanding of how the cell’s transportation network functions and how it responds to varying external conditions. Two measures that will help in understanding the microtubule network are angle direction and curvature. A distribution of angle direction of segments of microtubules will show the general direction of the microtubules. For instance, a travelling cell will have more microtubule segments oriented in the direction of travel. A distribution of curvature of the segments of microtubules will indicate changes in the orientation of segments of microtubules.

There is a large body of work studying the detection of the direction of ridges, edges, and lines; see [2]-[13]. Steerable filters are filters of any orientation created from linear combinations of oriented filters called basis filters [2]. Steerable filters can be used to determine the approximate orientation of an edge and can also be used to trace objects in images. For example, Yu *et al.* improved the accuracy of steerable filters using Gaussian functions as basis filters [6]. This resulted in directions that were more precise, and noisy edges were more distinguishable. In another paper, Jacob and Unser designed optimality criteria for steerable filters to detect edges, and published their software for use in a popular image analysis tool [4]. Steerable filters can work on edges with varied pixel widths, can determine the orientation of junctions with many branches, and can work for three-dimensional data. The returned orientation of an edge or junction is often dependent on the placement of the filter. As described by the above authors, a steerable filter may suggest, for instance, that a straight edge in an image is “bent” at an angle if the filter is centered beside the edge instead of directly on the edge. Because of this, centering the steerable filter is crucial to a reliable approximation of curvature and an issue we discuss further. Other work presents junction detectors, which find ridges or lines in an image that branch. Junction detectors play an important role in the study of the blood vessels and identify objects in an image. For instance, Tsai *et al.* and Sofka and Stewart present work on detecting junctions in retinal blood vessels to aid in the detection of low-contrast blood vessels and develop an automated technique due to the large quantity of images needing analysis [10] [11]. While microtubules have no junctions, two-dimensional images of overlapping microtubules appear to have many junctions where individual microtubules cross. In images of microtubules in the cell, determining the orientation of an individual microtubule can be complicated by two overlapping microtubules in cases where there is little sign on which microtubule went in which direction after a junction. In this way, junctions in microtubule images are an impediment. However we stress that we are not concerned with whole, individual microtubules. We instead focus on segments of microtubules, specifically the segments created between where microtubules overlap which appear as junctions in the two-dimensional image. Using segments of microtubules, our primary concern is for the structural properties of the entire network of microtubule segments. Thus we try to obtain a statistical understanding of the microtubule network found through analyzing the segments between junctions.

In this study, we propose a simple method to approximate the center of a microtubule by calculating average pixel intensity within a circle translated across the microtubule. This method has the benefit of being simple to understand conceptually. In the first part of this paper a method is developed to determine the center of a digital representation of fluorescing microtubules. With this center, an approximation for angle direction is possible. In the second part of this paper, we calculate curvature by finding three neighboring centers on a microtubule. Using both of these measures obtained from the method presented, we can better understand the organization of the microtubule network.

The goal of developing this method is to distinguish between microtubule network organizations under different cellular conditions, or to distinguish between different subpopulations of microtubules, such as those in which the tubulin subunits are post-translationally modified, by using a consistent and accurate technique for analyzing images of the microtubule network. The performance of the method will be studied using constructed “test” images with representations of microtubule-like structures in the form of circles, ellipses, and lines. The data collected from applying the method to these constructed microtubules can be compared to theoretical values to analyze accuracy.

There are different approaches to microtubule network structure. One is to map individual microtubules and collect appropriate statistics on direction. A second is to determine a statistical description of microtubule angles along with a probability density of microtubule presence. Here, an approach is adopted that obviates the need to map whole microtubules, but adds information on direction changes to the statistical approach. This is done by considering segments of microtubules and the distributions of angle direction and curvature of these segments. The probability of a microtubule with a determined direction is calculated within an analyzed area of the cell. This approach may produce a more rigorous, statistical analysis of microtubule network structure to determine any differences between different networks of microtubules.

## 2. Theoretical Foundations

Many situations involve spatial structures whose exact locations are of less concern than their density. In addition, structure directions are often of interest. Microtubules appear on the cellular scale as one-dimensional structures spanning large portions of a cell. Microtubule position and direction are determined by molecular scale processes such as dynamic instability, which could allow the structures to change in response to different conditions.

For the purposes of this study, we assume that a probabalistic description is appropriate. Thus, we seek a probability density function *P* (**x**,***τ***,***κ***), such that *P* (**x**,***τ***,***κ***) d**x**d***τ***d***κ*** describes the probability that a microtubule is found within d**x** of spatial point **x**, having direction within solid angle d***τ***, and having curvature within d***κ*** of ***κ***. The event space is the set of all cells subject to the same conditions.

The theoretical procedure for estimating probabilities is to sample many cells from the set of equivalent cells, locate the point **x** in each cell, and determine whether there is a microtubule within d**x** of **x** and, if so, determine if the direction of the microtubule is within d***τ*** of the direction ***τ*** and within d***κ*** of curvature ***κ***. Thus it is straightforward to consider the joint probability density function as a product of the absolute probability of microtubule presence *P* (**x**) and the conditional probability density function for direction and curvature, given that there is a microtubule at **x**. We have

P(x,τ,κ)=P(x)P(τ,κ|x).

There is much image data that is essentially two dimensional. In this case, the probability identity is

P(x,y,θ,κ)=P(x,y)P(θ,κ|x,y).

**Figure 1.**
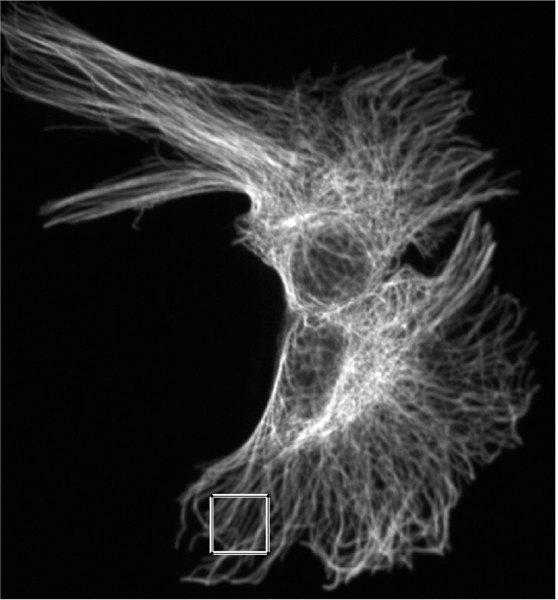
Combined images of fluorescing *α*-tubulin.

**Figure 2.**
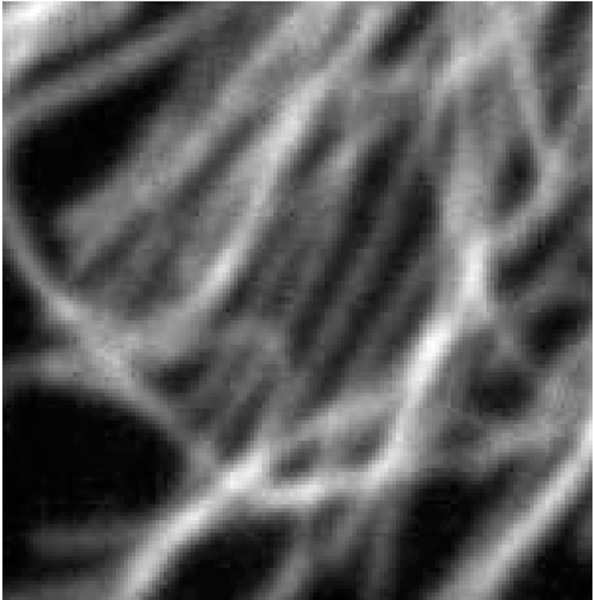
100 × 100 pixel image from outlined area.

Additionally, it is difficult to obtain samples of “equivalent” cells when the cells are not symmetric. In this case, we shall consider sub-areas of images that are nearly equivalent. For example, in Figure 1, the lower lune appears to contain microtubules that are equivalent in density and direction. One such sub-area is shown in Figure 2. The image in such a rectangular area centered at a point (*x*, *y*) and of size (Δ*x*, Δ*y*) can be described in terms of the pixel intensity *I* (*i*, *j*) at pixel (*i*, *j*), located at (*x* = *iδx, y* = *jδy*), where the pixel size is (*δx,δy*). Then

$$P(x,y)\approx v\sum_{i=i_{L}}^{i_{R}}\sum_{j=j_{B}}^{j_{T}}I(i,j),$$

where (*i*_*L*_, *i*_*R*_) and (*j*_*B*_, *j*_*T*_) delineate the rectangle of dimensions (Δ*x*, Δ*y*) centered at (*x, y*), and *v* is a normilization constant,

$$v=\frac{1}{\left(i_{R}-i_{L}+1\right)\left(j_{T}-j_{B}+1\right)I_{\max}},$$

where *I*_max_ is the maximum pixel value in the sample area.

What remains to be found is the conditional probability of microtubule direction *θ* and curvature ***κ***. In this paper, the method proposed obtains this probability by mapping microtubules in the image rectangle $\left(x-\frac{\Delta x}{2}<i\delta x<x+\frac{\Delta x}{2},y-\frac{\Delta y}{2}<j\delta y<y+\frac{\Delta y}{2}\right),$ while gathering statistics about direction and curvature.

## 3. Finding Points on Microtubules

Images of microtubules are two-dimensional grayscale images taken at several vertical positions through the cell (optical sections). Although the cell is three-dimensional, these cells are relatively flat (<5 μm tall). However, we typically take 8 – 15 optical sections per cell to maximize resolution, and then combine these layers using a maximum projection algorithm with image manipulation software such as Image J. Microtubules are labeled with a fluorescently tagged antibody to the tubulin subunit. The distribution of these antibodies along microtubules is generally considered to be uniform. These fluorescent molecules create the brighter pixel intensities shown in Figure 1 and Figure 2. One must remember that the images used are not actually images of microtubules, but fluorescent molecules attached to the microtubules. These molecules mark the location of microtubules like streetlights mark the location of roads in a satellite image. Each fluorescing body emits light from a central location in a gaussian distribution. Brighter areas of an image, therefore, are not typically the result of more fluorescent molecules on a microtubule in that area, but more likely represent several microtubules that are bundled in that area. One must also remember that what may look like a representation of a microtubule is actually several times larger than a microtubule because of the nature of fluorescence imaging.

### 3.1. Finding Points near the Center of Microtubules

Before finding a point near the center of the microtubule we first provide a loose definition of the center of a microtubule. Microtubules are cylindrical and a two-dimensional representation of them would allow for the center of the microtubule to be equi-distant from each side of the two-dimensional representation. However, the images we have of microtubules are, as mentioned elsewhere, actually images of fluorescents attached to tubulin subunits of the microtubule in an assumed uniform distribution. Because of this, the sides of the profile of a microtubule are difficult to define and thus so is the center in this way. Instead we define the center of a microtubule as it affects the curvature of that microtubule segment. A point is on the center of a microtubule if the curvature of the microtubule segment at that point is equal to the real curvature of the microtubule segment. For example, if the microtubule segment were straight and thus had a curvature of 0, a point on the microtubule segment is on the center if the angle between each direction (forward and backward) of the microtubule segment is π and thus the curvature would be 0.

**Figure 3.**
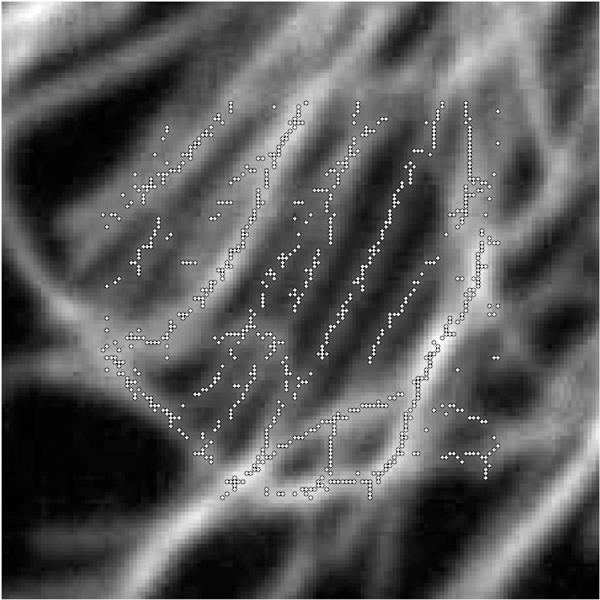
Initial points found for a region of microtubules using the described algorithm with *d* = 0.04 .

**Figure 4.**
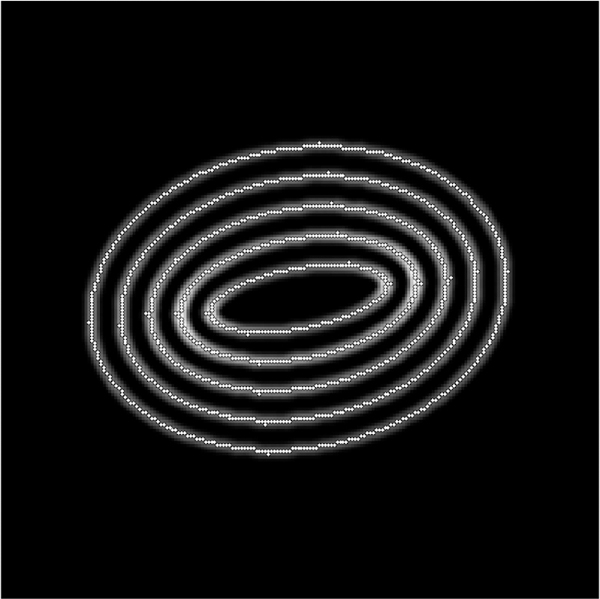
Initial points found for a test image using the described algorithm with *d* = 0.25 .

To find points on the microtubule that are close to the center, we find local maxima in the rows and columns of pixel data. These local maxima are at pixel locations with pixel intensity above that of neighboring pixels (in the row only or column only) by a specified amount *d.* Points found this way are then filtered so that no two points are within one pixel of each other; this avoids double counting at that pixel location. Using this process with a reasonable choice for *d* finds initial points on nearly all microtubules quickly. The process avoids picking initial points by hand which can create bias, or picking points randomly which often will not be well-centered on the microtuble. Figure 3 and Figure 4 show the initial points selected for a region of microtubules and a test image of ellipse shapes simulating microtubules (described in detail below).

From these points, we find points “centered” on the microtubule. We accomplish this by first finding the angle direction of the microtubule at that location and then translating a circle along the perpendicular angle direction. At many steps along this line, we calculate the average pixel intensity for pixels within the circle. We consider the “center”of the microtuble to be at the center of the circle with the highest possible average pixel intensity. We describe the technique used to find angle direction and the “centering” technique in more detail below.

### 3.2. The Centering Technique

To implement the centering technique, we first consider that pixel values can be mapped to integer locations on a Cartesian plane, where location is determined by the matrix location of the pixel in the image. For example, pixel (*i, j*) is located on the Cartesian plane at (*x, y*) = (*i, j*). On this plane, we can use geometric constraints like a circle with a radius of r pixels. In this way, we translate a circle across the width of a microtubule and calculate average pixel intensity at each step. The steps for this process are as follows:

1) Pick, as an initial point, a point with local maximum pixel intensity as discussed earlier.
2) From this initial point, calculate the angle direction of the microtubule.
3) Create a circle-shaped closed constraint centered on this point and translated perpendicular to the found angle direction through *n* steps in both directions. At each translation step, calculate the average pixel intensity within the circle-shaped closed constraint.
4) The center of the circle corresponding to the step with highest average pixel intensity is considered the center of the microtubule.
5) Repeat for a new initial point.

**Figure 5.**
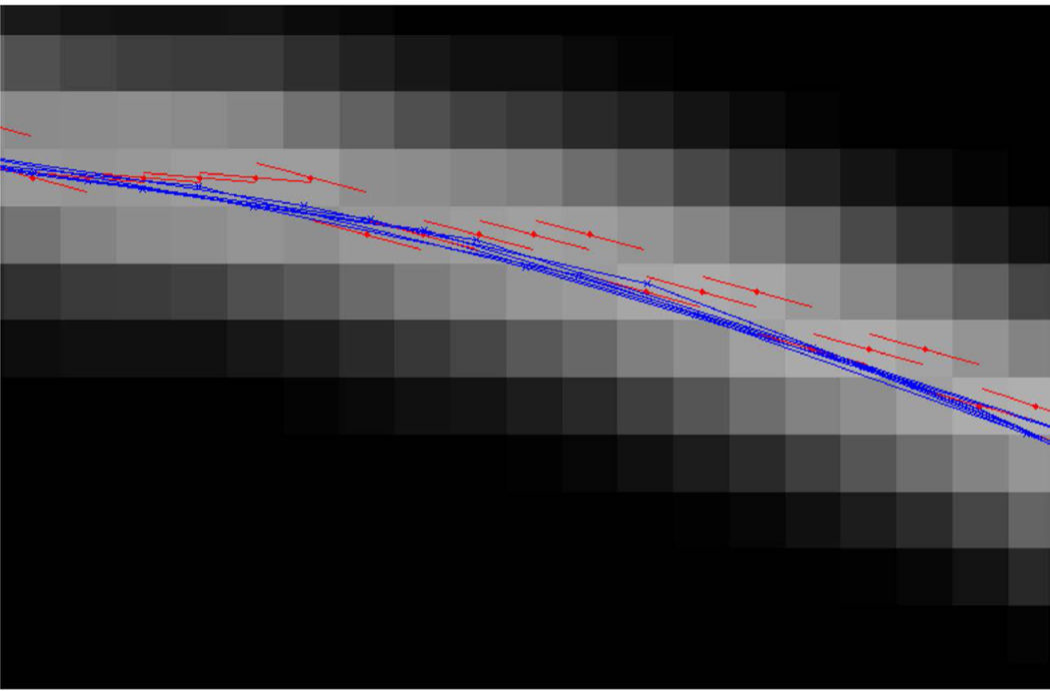
The centering technique finds more accurate locations of the curve center.Red dots show the pixel with locally maximum intensity and red lines show the angle direction found at those locations. Blue Xs show locations of the curve center found using the centering technique and blue lines connect triples of points to estimate curvature.

This process begins with an approximation to the microtuble center by first considering the local row and column maxima and then “centering” a circle on the microtubule, using angle direction and the circle as a guide. This process could be repeated multiple times by recalculating the angle direction at the new “center” and then re-centering the circle. For our proposes, we did not repeat the process. Figure 5 shows the results of this technique on several points.

From these centered points along the microtubule we are able to estimate curvature on test images. Implementing this technqiue requires two components not yet discussed: a way to calculate angle direction at a location, and a way to find neighboring triples of points on the microtubule for calculating curvature. To calculate angle direction, the steerable filters discussed above are a well-researched option. We explored another way to calculate angle direction and find neighboring triples of points using a technique similar to the centering technique described above.

### 3.3. Direction Angle

To measure the direction angle of a digital representation of a curve, we must measure angle based on the location of pixels that represent that curve. However measuring direction angle as the slope between two pixels representing points on a curve will result in discrete angles. We can achieve a higher accuracy by measuring the line, or curve, generated from multiple pixels. For images of microtubules, where the pixelated curve has a higher intensity in the center of the curve and lower intensity further from the center, we can consider a two-dimensional area of pixels to determine the direction angle of the curve at a point. We implemented a similar technique to the centering technique described above, using a rotated ellipse-shaped constraint instead of a translated circle. The angle determination method is as follows:

1) Pick an initial point on a microtubule.
2) Create a closed constraint shape centered on this point, and rotate the shape through angle π using *t* rotation steps. At each rotation step, calculate the average pixel intensity within the closed constraint shape.
3) The angle of rotation of the constraint shape corresponding to the highest average pixel intensity is considered the angle direction of the microtubule at the initial point.
4) Repeat for a new initial point.

**Figure 6.**
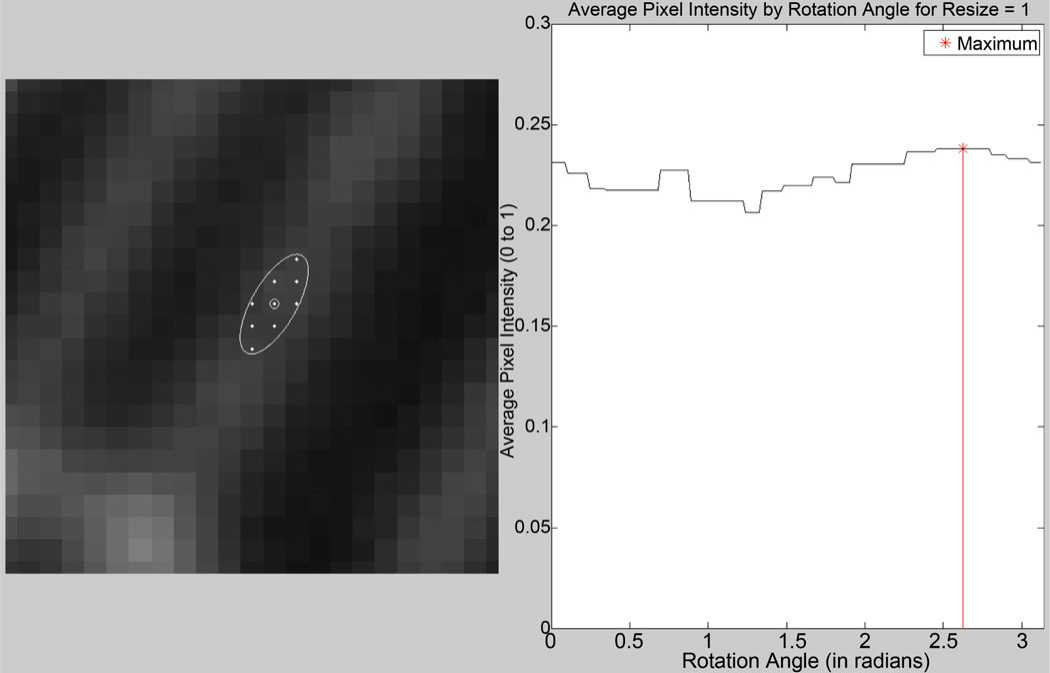
Best direction given by centered ellipse.

The rotated shape with the highest average pixel intensity will best fit over a given section of the fluorescing microtubule, distinguished by bright pixels in the image. See Figure 6.

The problem at each initial point can be written as an integer program:

$$\begin{array}{l} \underset{\theta }{\max}\sum p_{ij}b_{ij}\\ \;\text{s}.\text{t}.\;C\left(b_{ij},\theta \right)\leq 0,\\ b\in B \end{array}$$

where the *p*_*ij*_ is the pixel intensity at the image point (*i, j*), *b*_*ij*_ *=* 1 if the coordinate (*i, j*) satisfies the constraint and *b*_*ij*_ *=* 0 otherwise. The constraint *C* (*b*_*ij*_ ,*θ*) is a closed shape around a given starting point (*x*_*0*_*,y*_0_) and at an angle of *θ*. The closed ellipse-shaped constraint is determined by a single nonlinear contraint:

$$C:{\left(\frac{\left(y-y_{0}\right)\cos\theta +\left(x-x_{0}\right)\sin\theta }{m}\right)}^{2}+{\left(\frac{\left(y-y_{0}\right)\sin\theta -\left(x-x_{0}\right)\cos\theta }{n}\right)}^{2}-1\leq 0,$$

where the parameters *m* and *n* are chosen by trial and error so that the closed shape fits well over the microtubule without being too small or too large that data is inaccurate. The term *b*_*ij*_ is determined automatically in this case when the pixel at location (*x, y*) satisfies the constraint. This method cannot readily employ regular optimization program solvers, so the total pixel intensity of every rotation step must be found and then the angle with the largest corresponding average pixel intensity is chosen. In the result of a tie between nearby rotation angles, the average between the two maxima is taken.

**Figure 7.**
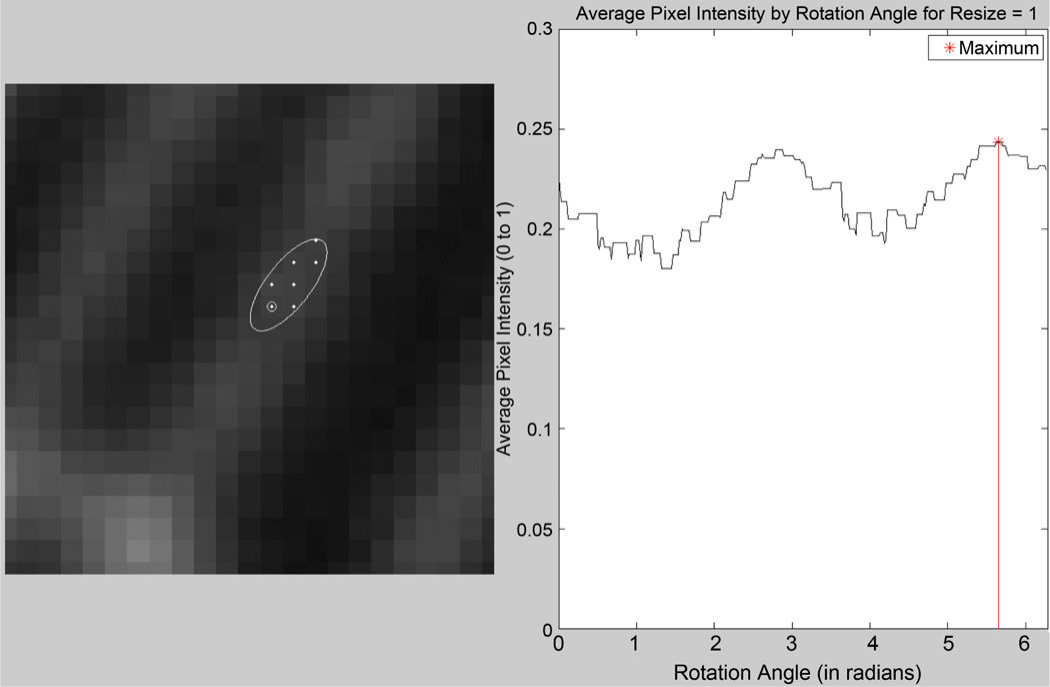
Best direction given by offset ellipse.

If the closed constraint *C* is offset from the initial point, instead of centered on it, then the resulting maximum average pixel intensity would indicate the approximate direction of travel of the microtubule (see Figure 7). In the thesis by DiLorenzo, using this technique as a means of “tracing” a microtubule is explored in more detail [14]. Here, we use the technique as a way of finding triples of neighboring points for calculating curvature.

## 4. Analysis of the Method Using Test Images

**Figure 8.**
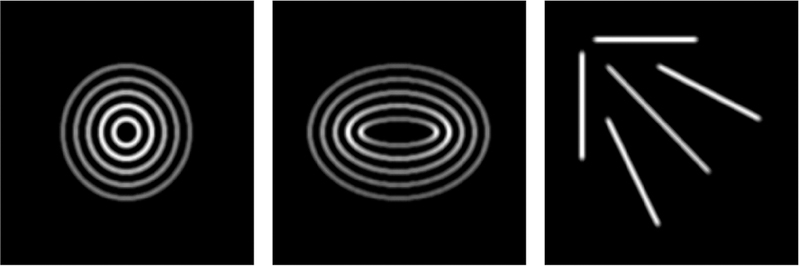
Test images of microtubules shaped like circles, ellipses, and lines.

We apply the method as described to several test images simulating microtubules following curves with known functions and curvatures: ellipses, circles, and lines. These test images are shown in Figure 8. The circle-shaped microtubules allow us to tune the method and verify accuracy. The ellipse-shaped microtubules allow us to test for invariance of the method under rotation and distinguish curvature distributions irom those of circles. The line-shaped microtubules allow us to test the effect of the image resize value on the method.

### 4.1. Resizing the Image for Better Accuracy

**Figure 9.**
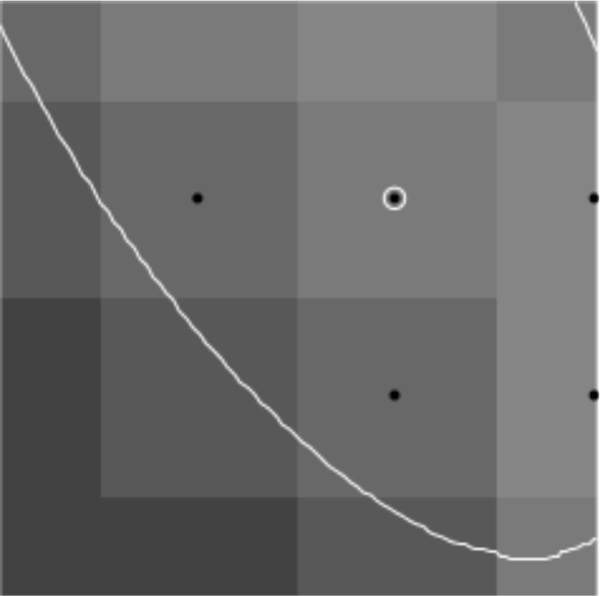
Resize of 1.

**Figure 10.**
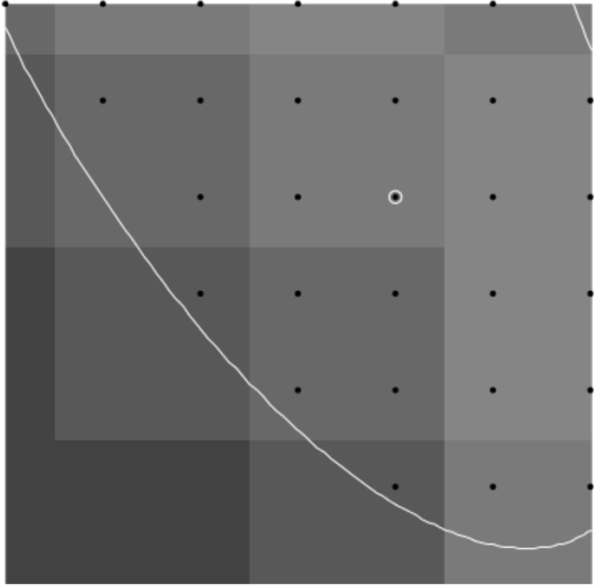
Resize of 2.

**Figure 11.**
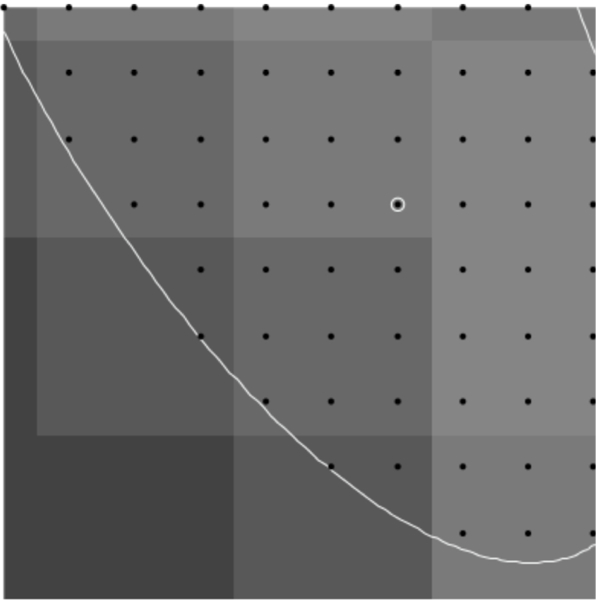
Resize of 3.

**Figure 12.**
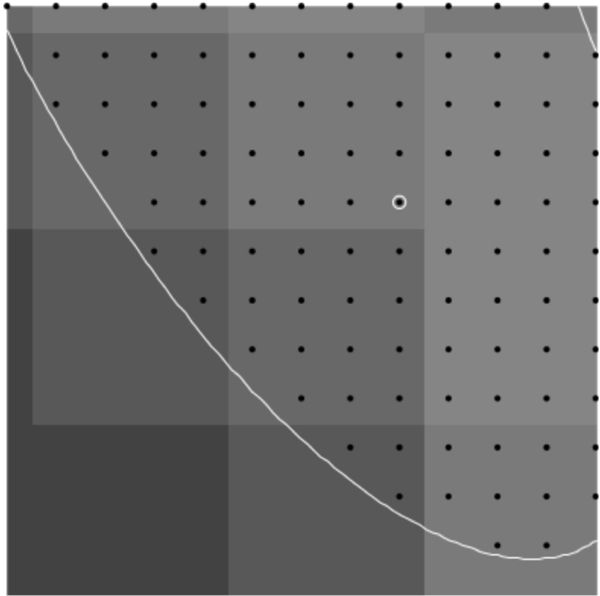
Resize of 4.

**Figure 13.**
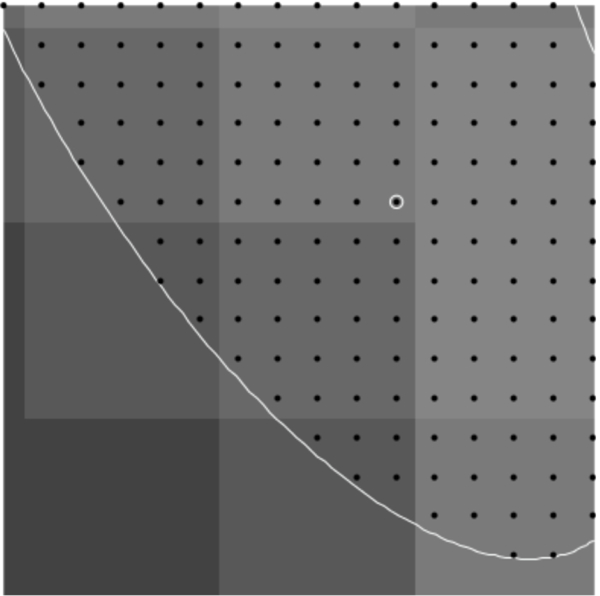
Resize of 5.

**Figure 14.**
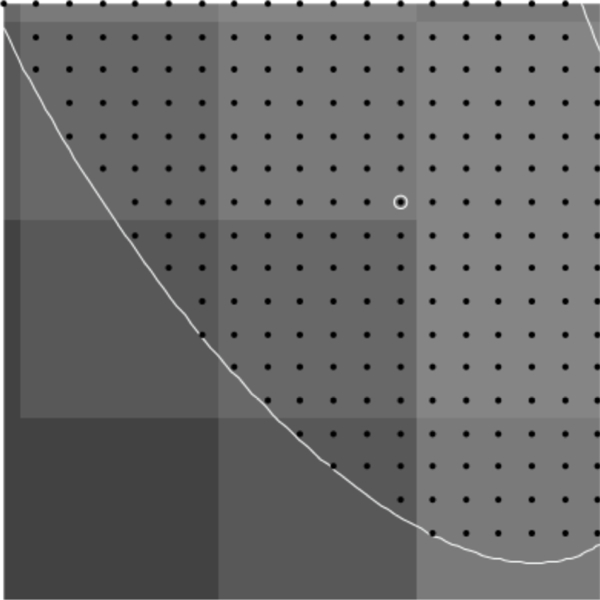
Resize of 6.

**Figure 15.**
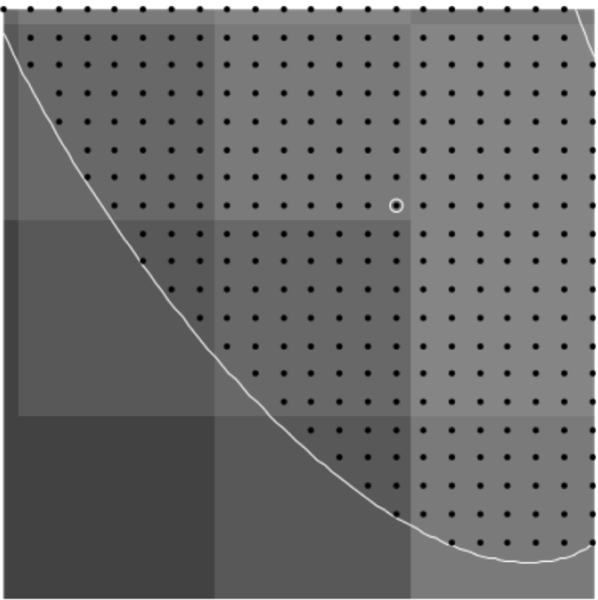
Resize of 7.

**Figure 16.**
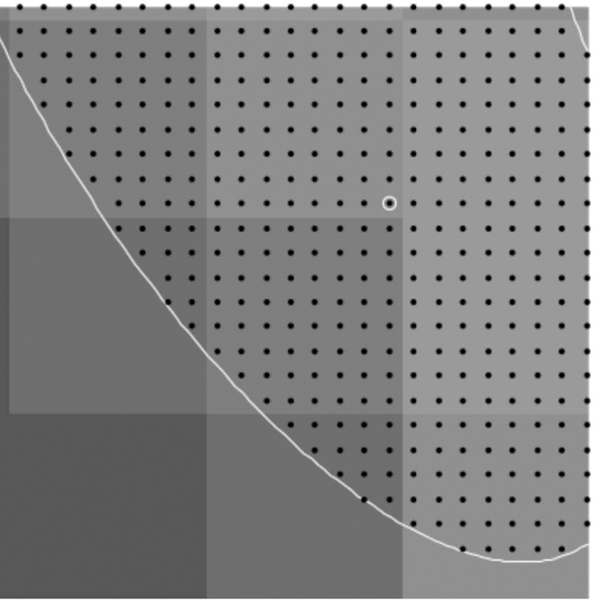
Resize of 8.

The constraints detailed above only consider intensities at integer points satisfying the constraint. Those points correspond to the location of a pixel in the image. However, the light entering the aperture of the microscope to be recorded as the pixel intensity can be associated with a two-dimensional area in the image, centered around the integer point. Because of this, the pixel value of an integer point can be used to represent the pixel value in a unit square area around the integer point. In this situation, the intensity of the entire square area is considered to be the pixel value at the integer point and the intensity of a fraction of the square area is assumed to be the same fraction of the intensity. For example, the intensity of half the square area is considered to be half the pixel value at the integer point. With a method that only considers integer points instead of area, the pixels near or on the border of the contraint will be entirely included or entirely excluded, when only a fraction of their pixel intensity is inside of the constraint. This issue creates a convex hull of the integer points that differs from the real shape of the constraint, and so could lead to inaccuracies in the direction chosen by the method. See Figures 9–16.

To represent the constraint with more accuracy, fractions of pixel values must be approximated near the border of the constraint. To accomplish this, the image can be resized so that each pixel intensity is represented by an *n* × *n* block of points distributed over the inside of the square. The contraint will then include some, but not all, of the resulting resized pixels in the new image. This represents a fraction of the original pixel intensity and allows a better approximation of the constraint. Resizing the image results in a longer computation time, so a trade-off between computation and accuracy is necessary.

### 4.2. Analysis of the Closed Constraint Shape Size

**Figure 17.**
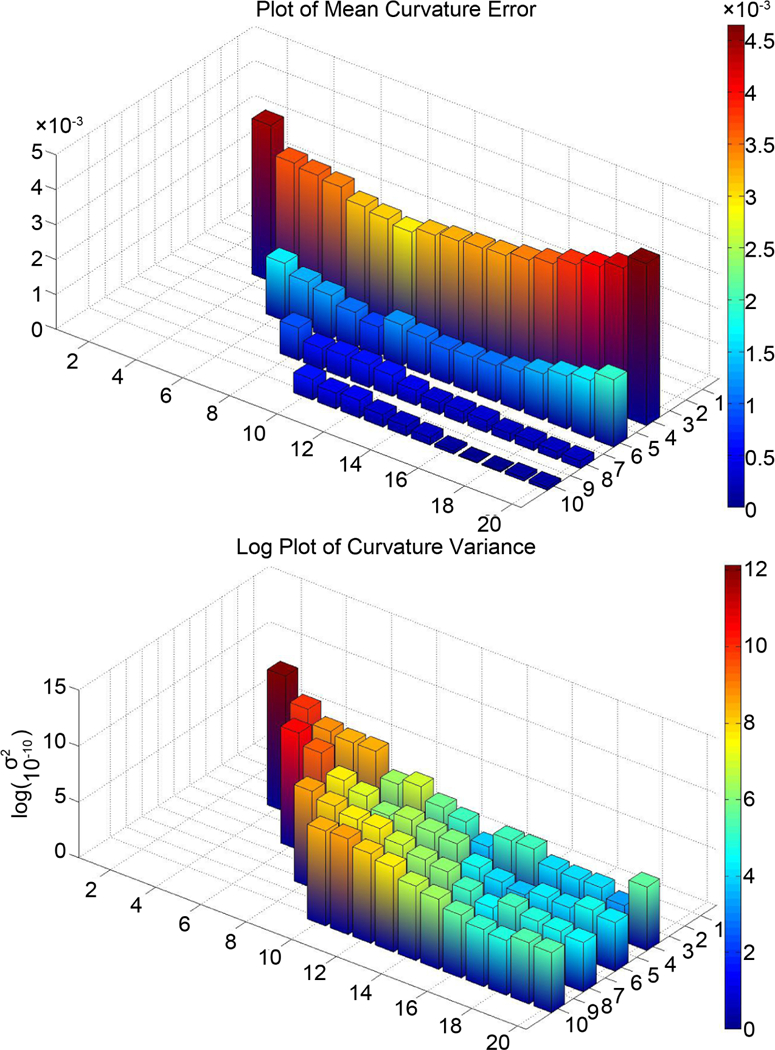
Mean error and scaled variance of the thirty-six starting points for different sizes of the ellipse-shaped constraint. The smaller axis is the minor axis and the larger axis is the major axis of the ellipse-shaped constraint.

Several parameters in the method affect the accuracy, consistency, or useability of the method. These parameters include the resize value, the size and shape of the closed constraint shape, the size of the increments by which the constraint is rotated, and the amount by the which the constraint is offset from the center of rotation. The size of the closed constraint shape (determined by parameters *n* and *m* in the constraint equation) were varied to study their effects on the method. To test for changes in the size of the ellipse constraint shape, the offset value, *δ*, was set to one-half the length of the major axis, while the lengths of the minor and major axes were varied from (4, 4) to (10, 20). A resize value of 64 was used with 36,000 rotation steps between 0 and 2π. We expect that a constraint shape too small will not accurately distinguish between similar optimal angle directions, while a constraint shape too large will “jump” from one microtubule to another. Larger constraint shapes require more computing time so a smaller shape is preferential. The largest of the five circle paths (with radius 50 pixels) is used to find curvature at the thirty-six starting points. The results are shown in Figure 17 for each constraint shape size. The figure on the top shows the error between the mean curvature of the starting points and the real curvature of 0.02. The figure on the bottom shows the variance between the starting points scaled so the differences can be seen better. In each graph, the axes represent the major and minor axes lengths of the ellipse constraint shape, where the major axis is always greater than or equal to the minor axis. Note that error decreases significantly when the ellipse is widened.

### 4.3. Analysis of the Resize Value

**Figure 18.**
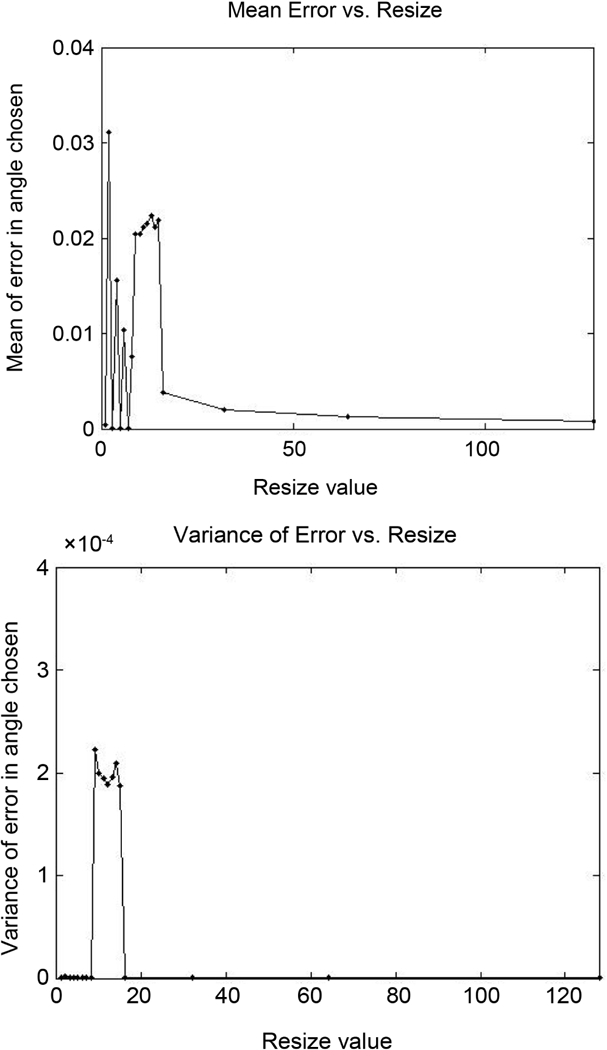
Mean error and variance of the twenty-six starting points along a line-shaped microtubule. The mean error generally trends downward with increasing resize value.

To test the improvements to accuracy of the method due to increases in the resize value, a test was designed to exploit starting points at fractions of a pixel. A line-shaped microtubule was used so that the method would have no variation between starting points other than pixel locations. The line used was the first line from the left in Figure 8. The first starting point was set at an integer coordinate, then twenty-five other starting points were chosen by keeping the x-position constant and varying the y-position in increments of 0.02 of a pixel. By doing this and using the two directions found for each starting point, we collect many results for each resize value. We expect that the directions found from the starting points will have more accurate means and lower standard deviations when the resize is high. The results of these tests are shown in Figure 18. Note that for odd resize values the mean error is zero, likely because the symmetry of the image helps the constraint center on the correct angle. An even resize value did not allow this. For other curves, this situation is unlikely to occur, and the error betwen successive resize values will likely be similar. When the resize value is increased, the mean error for resize values decreases. This shows an overall improvement when increasing the resize value.

### 4.4. Invariance Under Rotation and Translation

**Figure 19.**
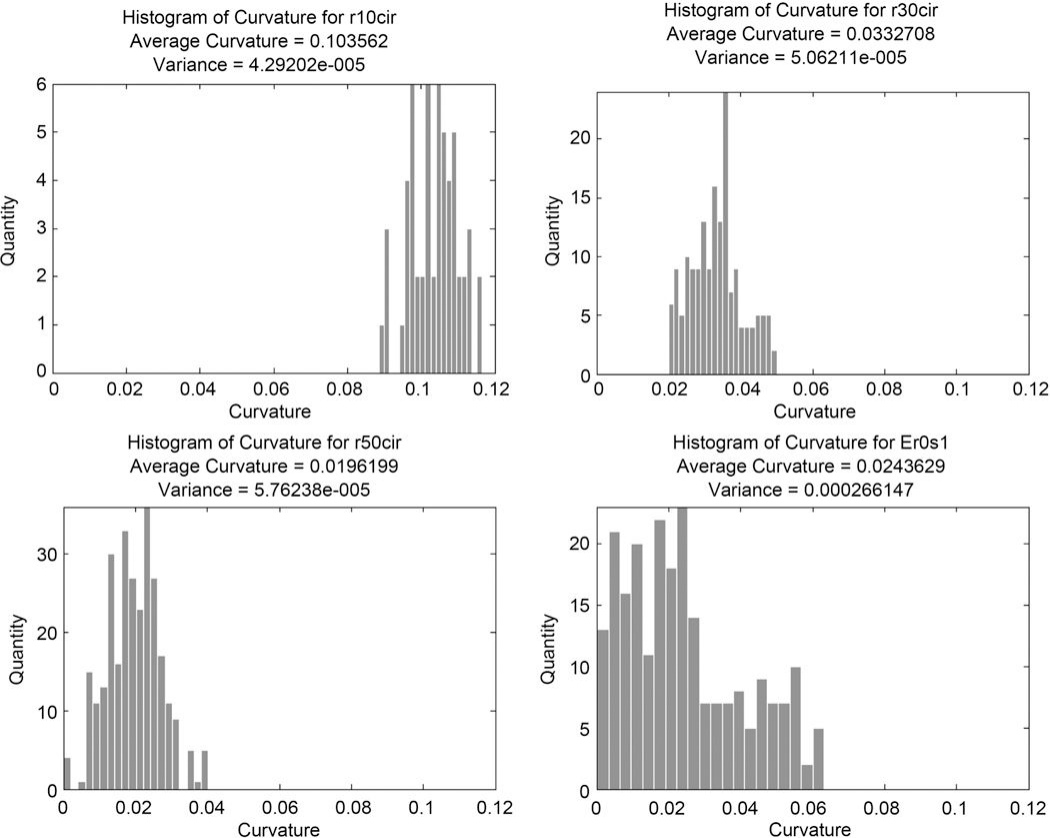
Distributions of curvature for circles of radius 10, 30, and 50 pixels, and an ellipse.

An important check for the method is invariance under rotation and translation of the image. To test for invariance under translation, the test image of circles was reproduced by shifting the generating equation by one-half of a pixel value, which will create a slightly different circle. To test for invariance under rotation, the test image of ellipses was reproduced by adding rotation to the generating equation and creating images rotated from 0 to π in increments of $\frac{\pi }{18}.$ We can conclude that the method is sufficiently invariant under translation and rotation of the image if the resulting data of these new images is similar to the corresponding data of the original two test images. Points are found and curvature is measured as described in the process above. A Kolmogorov-Smirnov test was used to check differences between two distributions. Examples of the resulting distributions can be seen in Figure 19.

With five circles, five translated circles, and five ellipses rotated eighteen ways, there are 4950 non-trivial comparisons of two distributions. Of these, 287 (5.80%) of the comparisons resulted in a false negative or false positive, given *α =* 0.05 . We notice that most of these errors occur from a lack of distinction between the two smallest ellipses. No errors occur due to changes in the rotation of the image, so we conclude that the method is sufficiently invariant under rotation.

Four of the five translated circles are indistinguishable from their counterparts, so we consider the method to be sufficiently invariant under translation.

**Figure 20.**
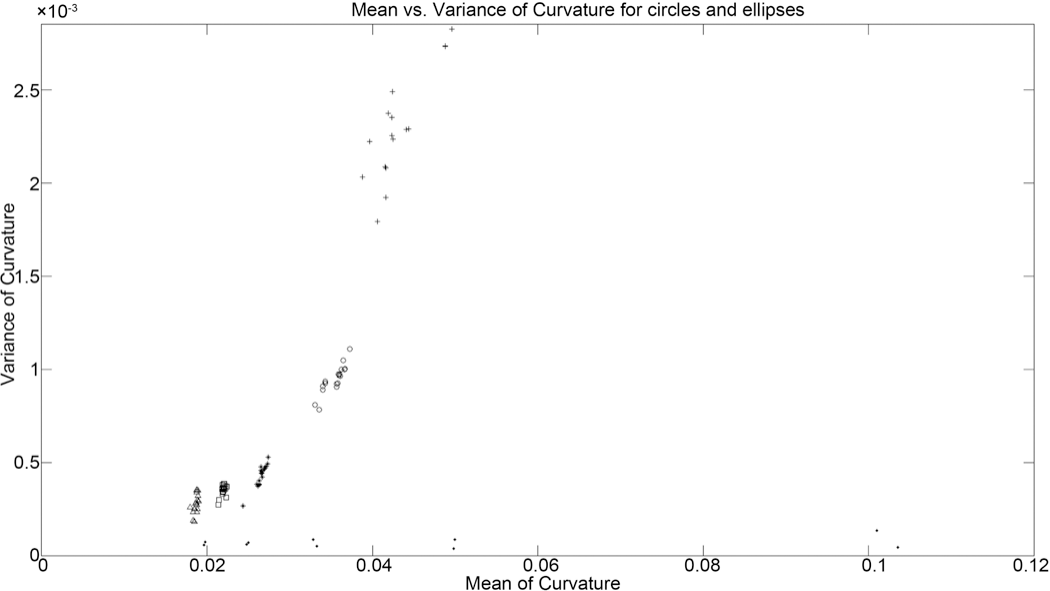
Mean vs. variance of curvature for all shapes. dots represent the ten circles tested. other symbols represent the differently sized ellipses.

Given that the curvature of a circle is constant and the curvature of an ellipse varies, we may consider only the means and variances of the curvature distributions as a way of separating different shapes. Figure 20 shows that for circles, translated circles, and ellipses of different rotations, the mean and variance of curvature are similar for similar shapes.

### 4.5. Independence of Curvature and Angle Direction Probabilities

Thus far we demonstrated that the proposed method for detecting angle direction and curvature at locations of digital curves works with reasonable accuracy and variability of the data. We also showed that the method produces similar results despite differing orientations of the same curve. For these results, angle direction and curvature are always considered separately. However, angle direction and curvature at a location may not be independent. For instance, in the ellipses shown in Figure 8, points on the curve with low curvature should also have angle direction closer to 0 than to $\frac{\pi }{2},$ and opposite for points with high curvature. For the circles in the same figure, we expect curvature to be independent of angle direction. Note that angle direction in these distributions was adjusted by subtracting the mean of the original data. By adjusting in this way, the distributions for rotated ellipses are similar, and so we see that the method gives independence of curvature and angle direction as we expect.

**Figure 21.**
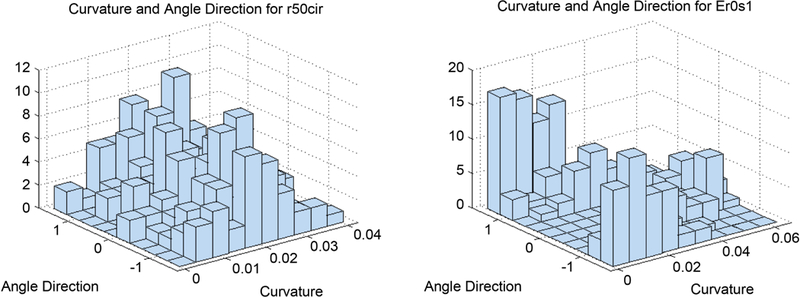
Distributions of curvature and angle direction for a circle of radius 50 pixels, and an ellipse.

Indepence of curvature and angle direction is important, as then the probability density function, P(θ,κ|x,y),can be computed as the product of the independent probability density functions: P(θ|x,y)and P(κ|x,y). The bivariate histograms in Figure 21 show these expectations are true for our method and suggest that the method accurately maintains indepence of curvature and angle direction.

## 5. Analysis of Data from a Section of Microtubules

**Figure 22.**
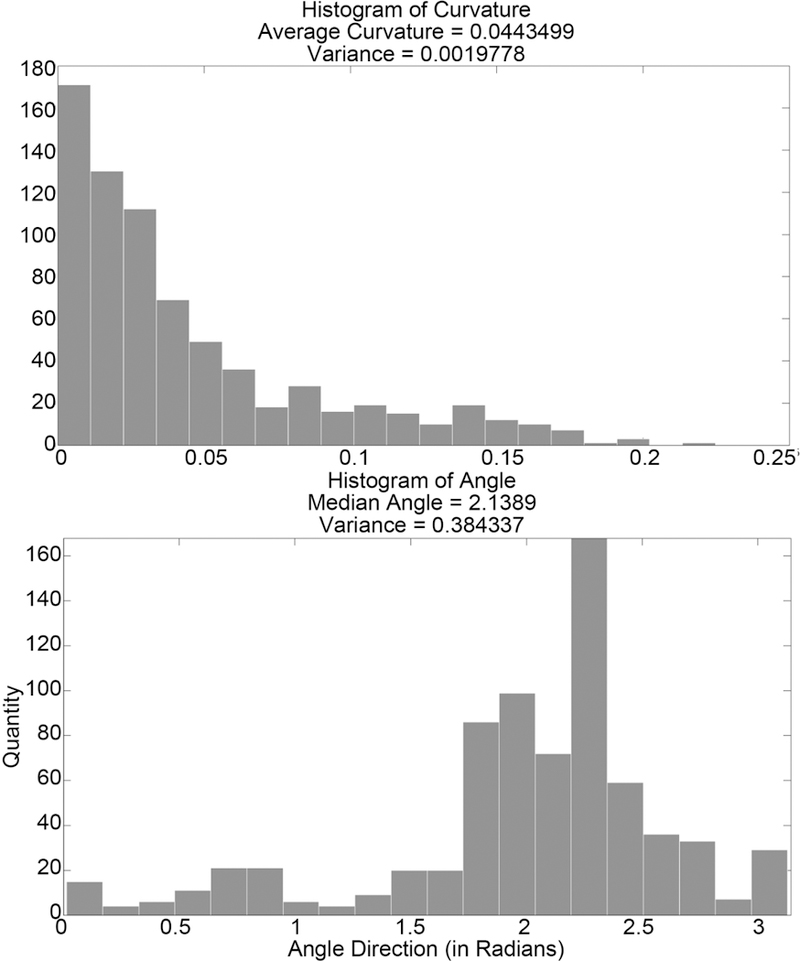
Histograms of curvature and angle distributions found for the section of microtubules.

With an understanding of the performance of the method, we can apply the method to the section of microtubules shown in Figure 2. Initial points are found as described, resulting in the points shown in Figure 3. These points are centered and matched with neighboring points to create triples. In rare instances, the triples will form an angle too sharp to consider possible for real microtubules. Because of this, triples that form an angle over $\frac{\pi }{3}$ are removed from the results. The resulting distributions of curvature and angle direction are shown in Figure 22.

**Figure 23.**
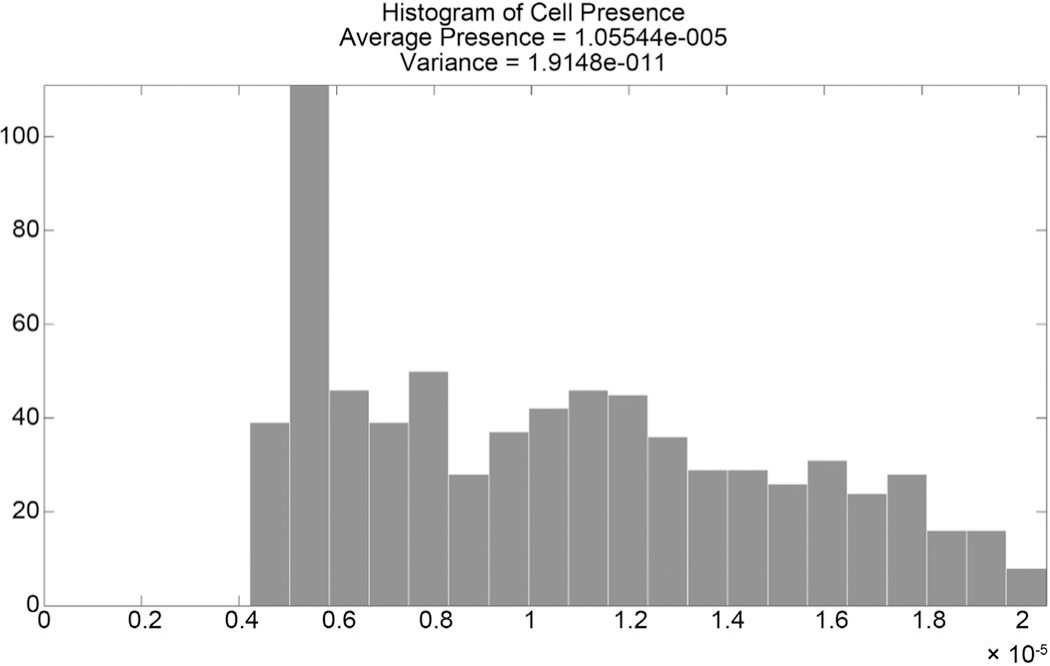
Histogram of microtubule presence.

**Figure 24.**
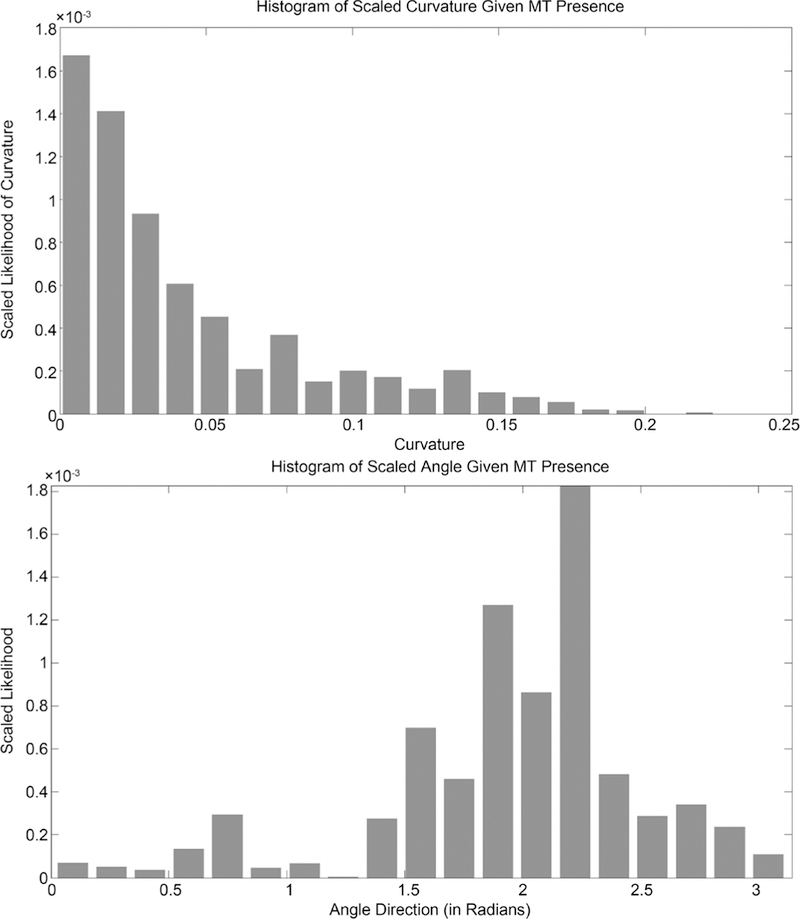
Histograms of scaled curvature and angle distributions given microtubule presence.

The distribution of curvature is what we use as the conditional probability density for curvature, given microtubule presence. Applying the probability of microtubule presence as described to this distribution creates the probability of microtubule curvature at a location. The probability density of microtubule presence is shown in Figure 23 and the resulting probability density of microtubule curvature is show in Figure 24. These distributions are independent are thus their product gives the probability of a microtubule having a given curvature and angle direction at a location. Distributions from sections of microtubules as shown here may be compared using the Kolmogorov-Smirnov test, by simply comparing means and variances, or other tests.

## 6. Conclusion

This method maps the high pixel intensity values of an image and reliably records microtubule curvature except at crossings. The resulting data may be used to show a distinction between microtubule subsystems, just as it shows in this study a distinction between several simple paths. This method can be used to determine the orientation of segments of the microtubule network and then to analyze changes in the microtubule network during events such as cell motility or wound repair. This method can also map, find angle direction, and find curvature of other networks such as blood vessels in the eye, tree limbs, hiking trails, roads, and river deltas. Parameters will need to be adjusted to suit the image and expected properties of the network to ensure accurate results.

Throughout the above research, one issue is mentioned repeatedly that would produce inaccurate results for our purposes if not resolved. This is the issue of finding the center of ridges, or microtubules in our case. In [4] [6] [7] [9], implementing the solution at the incorrect center of the ridge or junction produces skewed results. We employed a simple centering technique that reduces much of the variation in curvature for our test images.

The parameters of this method were chosen during testing to ensure more accurate results, but more analysis of the parameters is needed to find a best combination. Most notably, the resize value, fineness of the rotation step, the parameters determining the width and length of the constraint ellipse and the offset value play a crucial role in gathering useful data. The width and length of the constraint ellipse were varied to study the data gathered on the circles of the test image, but the amount by which the ellipse was offset from the current point was not varied. Empirically, the major axis of the ellipse played a major role in generating poor results when too large, while the minor axis of the ellipse helped generate better results when larger.

With this method as a starting point, we hope to establish a process for calculating the curvature of a digital curve at a specified loction along the curve. Several issues must be resolved to create a more useful and reliable method. One of these is computation time associated with a high resize and a fine mesh for the rotation steps. Another issue is determining the change in arc length that the closed constraint shape uses to find the corresponding change in angle. Lastly, we must study the differences between the true curve and the digital representation of the curve, in relation to choosing initial points for the method. This method can be used for three-dimensional situations, such as structures of cells, after changes to the structure of the closed constraint shape and the search area are made.
